# Supplementary material for: Causal Relationship between Aging and Anorexia Nervosa: A White-Matter-Microstructure-Mediated Mendelian Randomization Analysis
Source: Biomedicines. 2024 Aug 16;12(8):1874. doi: 10.3390/biomedicines12081874 (PMC11351342; doi:10.3390/biomedicines12081874)
Supplement: Supplementary file 1 [file biomedicines-12-01874-s001.zip › Supplementary Figure/Figure S8/Leaveoneout plot of MR analysis for TL to ubm-b-2019.pdf]

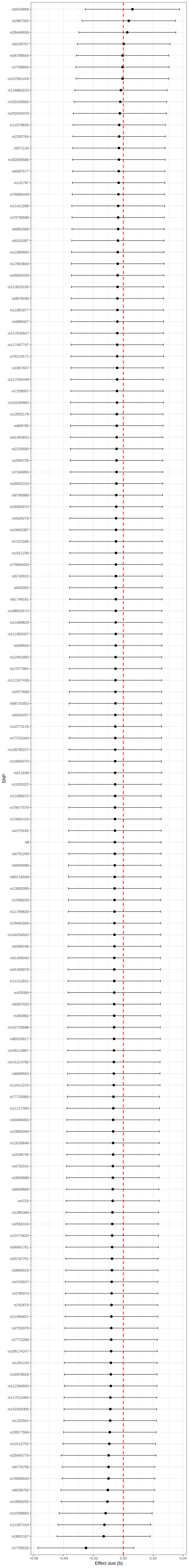
SNP
Effect size (b)
-0.06
-0.04
-0.02
0.00
0.02
0.04
rs9419958
rs2967355
rs35446936
rs8105767
rs56799554
rs7790856
rs137901416
rs116863223
rs150150565
rs202034370
rs11579626
rs2282764
rs871134
rs182059586
rs6587577
rs131797
rs76666449
rs11412296
rs73730598
rs6881568
rs8102497
rs12369950
rs17803849
rs45604339
rs113525195
rs9878436
rs11991877
rs4695407
rs117630647
rs117407747
rs76219171
rs1957937
rs117034449
rs7209057
rs143190905
rs12932179
rs869785
rs41304832
rs2230590
rs2056726
rs7164950
rs28502153
rs6790988
rs28363070
rs4530278
rs10645387
rs7221585
rs1611236
rs75664430
rs5742915
rs932002
rs61748181
rs188918174
rs11699829
rs111950327
rs939916
rs12451892
rs17677991
rs111527438
rs2977608
rs66731853
rs6054257
rs10773176
rs77231040
rs139795227
rs10840270
rs611646
rs1003322
rs11085072
rs79977579
rs73581419
rs2276182
All
rs6751209
rs9940099
rs80116508
rs13062095
rs7099229
rs11769630
rs78491606
rs144204502
rs9398196
rs61405042
rs41269079
rs11212631
rs429358
rs6007020
rs450962
rs142730696
rs80324517
rs145114957
rs141214782
rs6669563
rs12412214
rs77732866
rs11117354
rs59409453
rs10805346
rs13230646
rs2538745
rs4731541
rs3093888
rs6659669
rs4724
rs1985369
rs2555104
rs10774624
rs56061761
rs55747751
rs9600019
rs4743037
rs3785074
rs762679
rs11584821
rs2763979
rs7772289
rs185174247
rs1291143
rs16978028
rs112394943
rs117512405
rs142426306
rs1332941
rs28577594
rs10112752
rs35640778
rs6776756
rs76065543
rs6536702
rs10905255
rs10768683
rs11557154
rs3891167
rs7705526
